# The Traditional Uses, Phytochemistry and Pharmacology of *Sarcandra glabra* (Thunb.) Nakai, a Chinese Herb With Potential for Development: Review

**DOI:** 10.3389/fphar.2021.652926

**Published:** 2021-04-22

**Authors:** Yuanlian Zeng, Junyu Liu, Qiang Zhang, Xuhua Qin, Zulun Li, Guojuan Sun, Shenrui Jin

**Affiliations:** ^1^ College of Pharmacy, Chengdu University of Traditional Chinese Medicine, Chengdu, China; ^2^ International Department of Gynecology, Hospital of Chengdu University of Traditional Chinese Medicine, Chengdu, China; ^3^ College of Basic Medicine, Chengdu University of Traditional Chinese Medicine, Chengdu, China

**Keywords:** *Sarcandra glabra* (Thunb.) Nakai, traditional uses, phytochemistry, pharmacology, toxicity

## Abstract

*Sarcandra glabra* (Thunb.) Nakai is a folk medicine with a long history in China, which has been applied to treat sore throat, abscess, even tumor and so on. Meanwhile, it is also used as tea in some areas. At present, more than 200 chemical compounds have been isolated and identified from it, such as, sesquiterpenes, flavonoids, phenolic acids, coumarins and so on. Pharmacological studies have already confirmed that the extracts of *S. glabra* have many effects, such as antibacterial, antiviral, anti-inflammatory, anti-tumor, and anti-thrombocytopenia, especially the effects of anti-tumor and anti-thrombocytopenia are confirmed in clinic. Therefore, this paper systematically summarized the traditional uses, botany, phytochemistry, pharmacology, and toxicity of *S. glabra*, in order to provide a beneficial reference of its further research.

## Introduction

*S. glabra* is a perennial evergreen plant belonging to the Chloranthaceae family, and its resources are widely distributed throughout China, Japan, Korea, and Southeast Asia (Xu et al., 2011). *S. glabra* is commonly called *Zhong Jie Feng* in Chinese, because its ripe fruits resemble shiny red coral beads, it is also known as *Cao Shan Hu*. Meanwhile, after soaking *S. glabra* in hot water for a period of time, it will emit attractive aroma and taste delicious. Therefore, it is also regarded as tea in some areas (Yang, 1992; Han et al., 2013), also known as *Jiu Jie Cha*.

**FIGURE 1:**
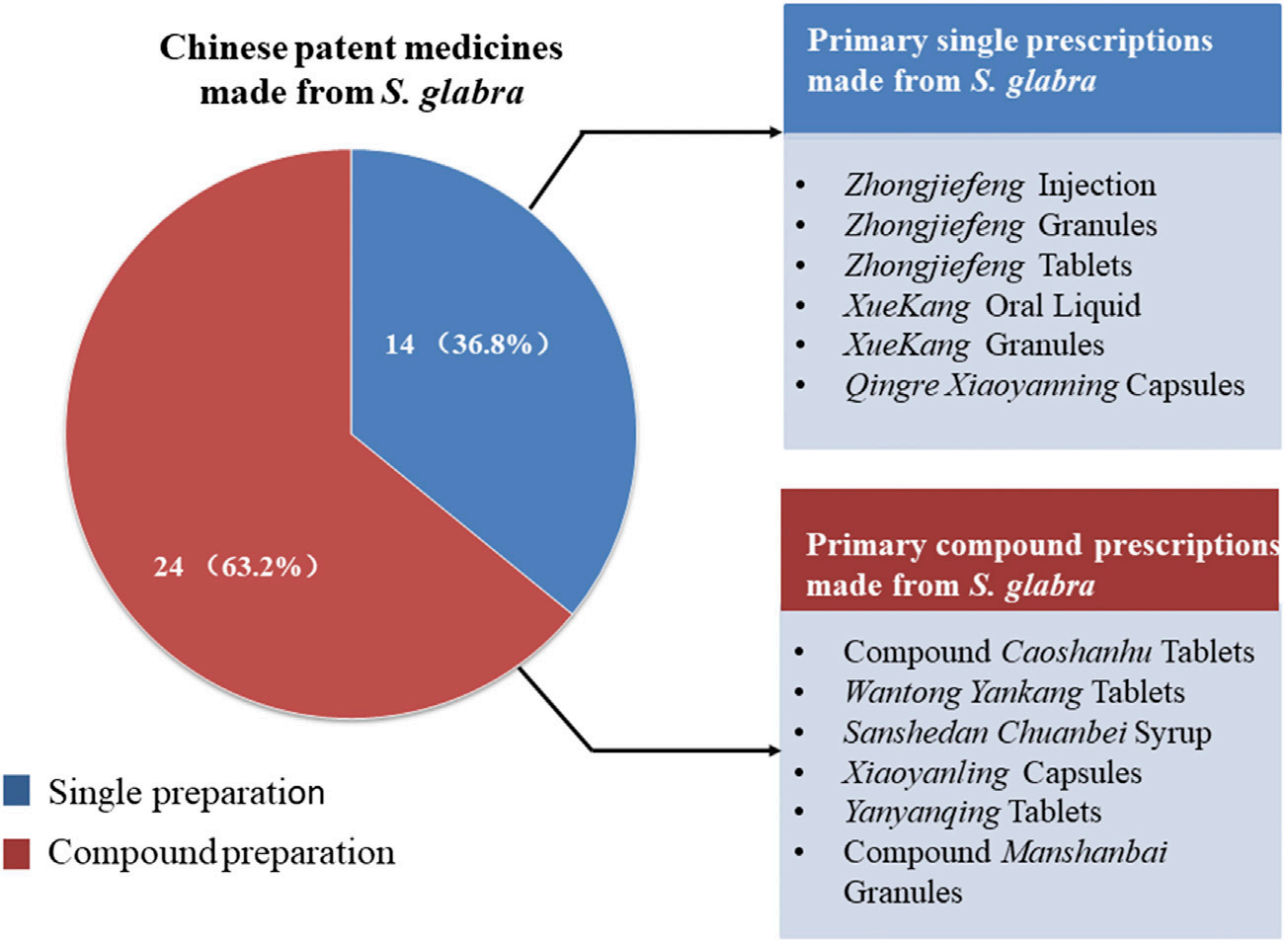
Chinese patent medicines made from *S. glabra*.

*S. glabra* has high medicinal value. *S. glabra* has been used as a folk medicine since the Qing Dynasty (Chen and Li, 2015), commonly applied by numerous ethnic groups in clinical practice in China, such as Han, Miao, Dong, Yao, Zhuang, *etc*., which has been officially listed in *Chinese Pharmacopoeia* since 1977. Traditionally, *S. glabra* is widely used to treat traumatic fracture, joint swelling and pain, sore throat, abscess, bleeding, and other diseases (Jia and Li, 2005). In modern clinical practice, it also has been applied to treat upper respiratory tract infection (Li, 2003), pneumonia (He et al., 2003), gastritis (Chen et al., 2012), viral myocarditis (Li, 2004), tumor (Cong et al., 2005; Song, 2017), and thrombocytopenia (Jiang and Zhou, 2003; Su and Luo, 2009), with significantly clinical therapeutic effect. Owing to the advantages of definite clinical effect, good safety, and abundant resources, many Chinese patent medicines with *S. glabra* as primary ingredient have been developed in modern times, 38 kinds of which have been approved for marketing by the State Food and Drug Administration of China (Figure 1).

In recent decades, considerable work has been done on pharmacology and phytochemistry of *S. glabra*. Many studies have proved that *S. glabra* exhibits a plenty of pharmacological effects, such as anti-inflammatory (Tsai et al., 2017), antibacterial (Jiang et al., 2000), antiviral (Cao et al., 2012), anti-tumor (Zhang et al., 2014), antioxidant (Liu et al., 2016), and anti-thrombocytopenic effects (Lu et al., 2018b). So far, over 200 chemical compounds have been isolated from *S. glabra*, including sesquiterpenes, flavonoids, coumarins, phenolic acids, lignans, anthraquinones and steroids. Among them, flavonoids are considered to be important bioactive components in *S. glabra*, which are also closely related to anti-thrombocytopenic activity of *S. glabra* (Xu et al., 2005). However, findings on pharmacology and phytochemistry are still difficult to comprehensively reflect its pharmacological effects and mechanisms, most pharmacological studies are still focused on exploring the activity of crude extracts, and the correlation between pharmacological effects and chemical components has yet to be fully established. Thus, there are many issues that deserve further investigation.

At present, reviews on *S. glabra* are not comprehensive enough (Han and Wu, 2017; Yang, 2017), and the chemical constituents and mechanism of pharmacological effects are deficiency, which impedes further research of *S. glabra*. In this paper, we used “*Sarcandra glabra*” as the keywords to collect information related to *S. glabra* from Web of Science, Science Direct, Springer, Google Scholar, PubMed, China National Knowledge Infrastructure (CNKI), and other professional websites, as well as classic books of herbal medicine. This paper intended to make a comprehensive and systematic review about *S. glabra*, so as to enhance further understanding of its traditional uses, botany, phytochemistry, pharmacology, and toxicity. This paper would also provide a beneficial reference for its in-depth research, development and utilization.

## Botany

The genus *Sarcandra* comprises three accepted species worldwide (Chen and Cheng, 1994). *Sarcandra glabra* (Thunberg) Nakai is a species of the genus *Sarcandra*, widely distributed in the south of the Yangtze River in China, as well as other Asian countries, including Korea, Japan, Malaysia, Philippines, Vietnam, India, *etc.* (Zhou, 1993; Chen and Cheng, 1994). It is a semi-shade plant, prefers a warm and humid environment, but avoids direct sunlight, thus, it usually grows in ravines, slopes, valleys, and wet places under forests.

**FIGURE 2:**
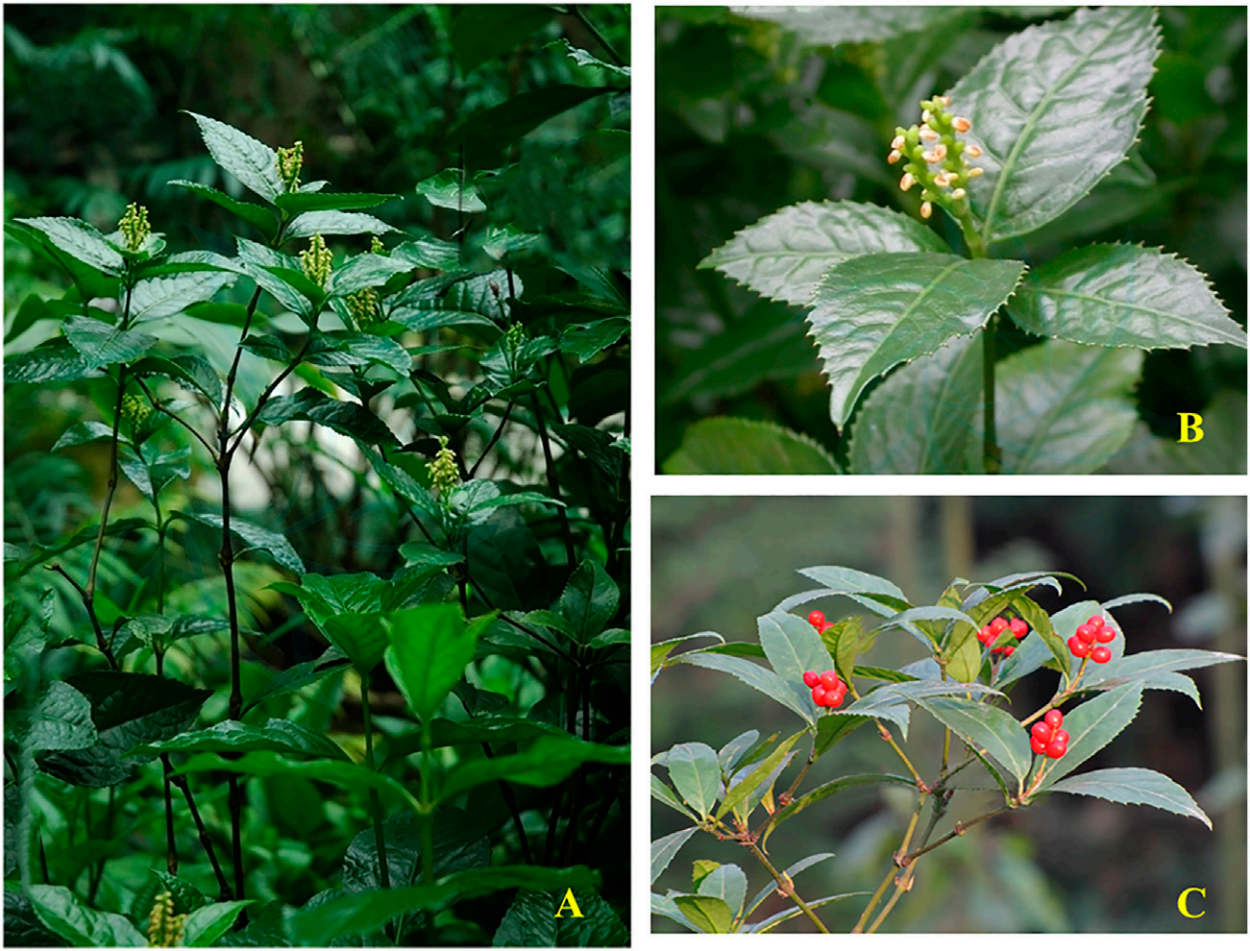
*Sarcandra glabra.* **(A–C)** represent the whole plants **(A)**, inflorescence **(B)** and fruits **(C)** of *S. glabra*.

*S. glabra* derives from the dried whole plant of *Sarcandra glabra* (Thunb.) Nakai (synonym: *Chloranthus glaber* (Thunb.) Makino), which belongs to the genus *Sarcandra* of the Chloranthaceae family. It is a perennial evergreen subshrub with a height of approximately 50–120 cm. Its stem is erect, usually branched, and the nodes of the stem and branches are obviously swollen, which also have obvious longitudinal grooves and ridges between the nodes. Its leaves are opposite, leathery or papery, and glabrous on both surfaces. The shape of leaves is ovate or oval, about 6–17 cm long and 2–6 cm in wide. Its leaves are similar to tea leaves, the apex is acuminate, the base is wedge-shaped, the edges are serrated, and the marginal teeth are hard bone. Its petiole is approximately 1 cm in length. The stipule is small, like a sheath. There are small yellow-green flowers on the top of the branches, with a fragrant smell, no perianth, and cluster into spikes. *S. glabra* is monoecious, in which the stamens are clubbed to cylindrical, while the pistil is globose. Its fruit looks like pearl, which turns into shiny red at maturity, about 3–4 mm in diam. The florescence ranges from June to July, and the fruit period is from August to October. The whole plant of *S. glabra* is shown in Figure 2 [(Cheng, 1982), http://ppbc.iplant.cn/sp/15108].

## Traditional Uses

*S. glabra* was first found in the Tang Dynasty’s medical book “*Ben Cao Shi Yi*” (AD 741) under the name of *Jie gu mu*, and then it was recorded in the Ming Dynasty’s Plant book “*Ru Nan Pu Shi*” (AD 1620) in the name of *Shan hu* (Chen and Li, 2015). However, its medicinal value was first appeared in “*Sheng Cao Yao Xing Bei Yao*” (AD 1711) in the Qing Dynasty: “Boiling it in water to drink, reducing fever”. According to “*Ben Cao Gang Mu Shi Yi*” (AD 1765), *S. glabra* could treat traumatic injury and fracture. In traditional clinical practice, *S. glabra* was effective in the treatment of joint swelling and pain, sore throat, carbuncle, tumor, trauma, bleeding, *etc.* In particular, the production technology of Miao nationality using *S. glabra* to treat traumatic fracture has been included in the list of National Intangible Cultural Heritage Protection at present (http://www.ihchina.cn/). Furthermore, in both ancient and modern times, *S. glabra* has been commonly used by Miao nationality to treat postpartum abdominal pain and dizziness; Dong nationality to treat appendicitis; the nationality of Yao and Zhuang to treat stomachache, dysentery, and influenza; Dai nationality to treat gastric ulcer; the nationality of Jinpo and Lahu to treat many gynecological diseases such as irregular menstruation, dysmenorrhea, and puerperal metrorrhagia (Jia and Li, 2005).

*S. glabra* has long been regarded as an edible plant in some areas. According to the records of Xingan County Chronicles in Jiangxi Province, people grind *S. glabra* with salt, rice, sesame and houttuynia in pottery bowls, then mix it with well water to drink, which is locally called *Lei Cha*. *Lei Cha* has been popular in the region since the Ming and Qing Dynasties, at present, *Lei Cha* in Gannan of Jiangxi Province has been included in the list of National Intangible Cultural Heritage Protection (http://www.ihchina.cn/). The Dong, Miao, Shui, Buyi and other ethnic groups in Guizhou province use *S. glabra* to make tea instead of ordinary tea in daily life. Especially, the Dong people prefer to make camellia oleifera for consumption through mixing *S. glabra* tea with glutinous rice, peanuts, soybeans and other condiments they like. Hence, the above records illustrate the safety of *S. glabra* as a medicine from another point of view.

## Phytochemistry

**FIGURE 3:**
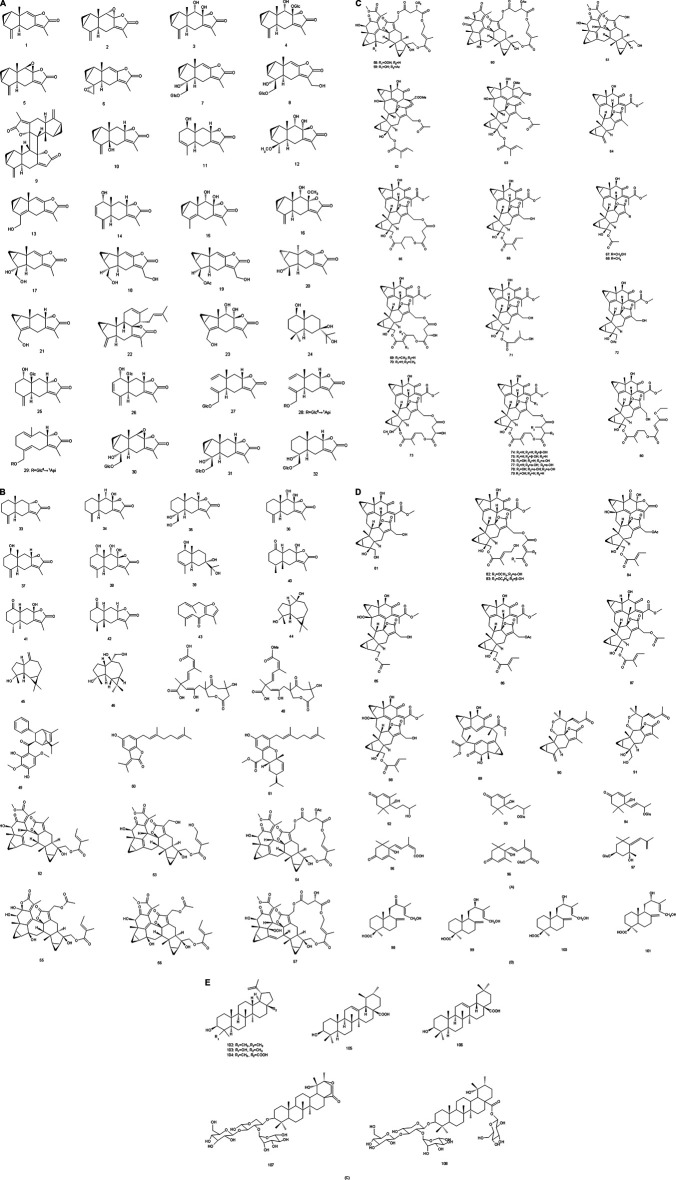
**(A)** Chemical structures of sesquiterpenes **(A)** identified in *S. glabra* extract. **(B)** Chemical structures of sesquiterpenes **(A)** identified in *S. glabra* extract. **(C)** Chemical structures of sesquiterpenes **(A)** identified in *S. glabra* extract. **(D)** Chemical structures of sesquiterpenes **(A)**, and diterpenes **(B)** identified in *S. glabra* extract. **(E)** Chemical structures of triterpenes **(C)** identified in *S. glabra* extract.

**FIGURE 4:**
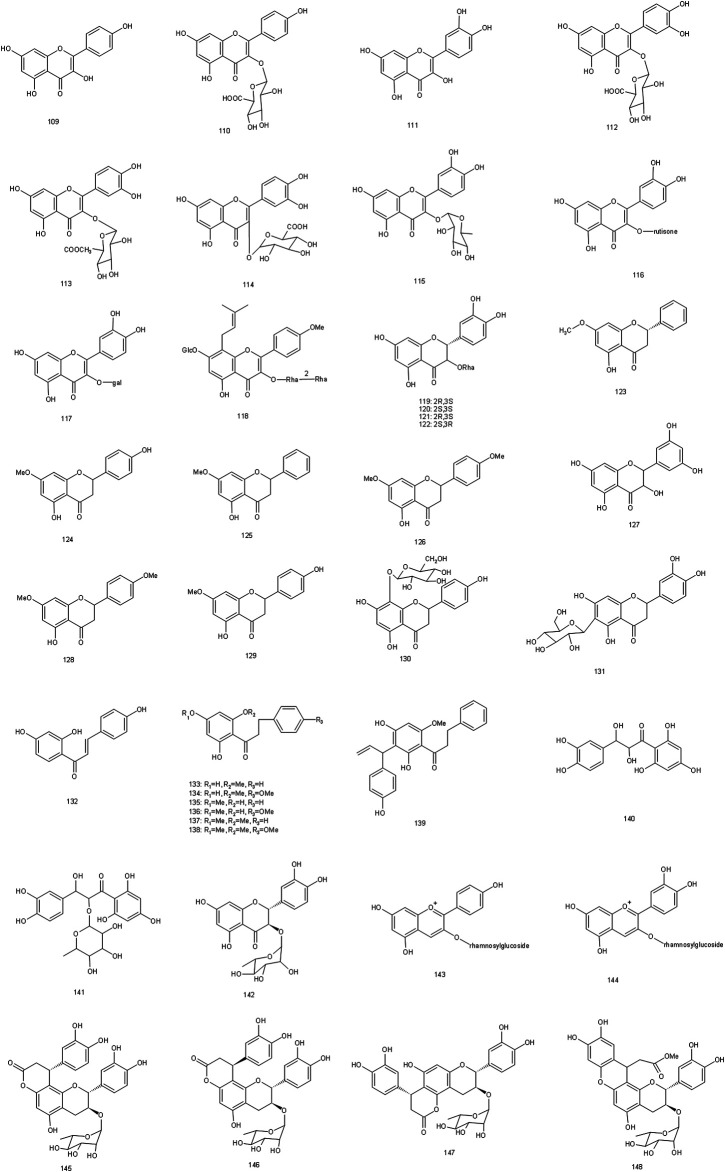
Chemical structures of flavonoids identified in S. glabra extract.

**FIGURE 5:**
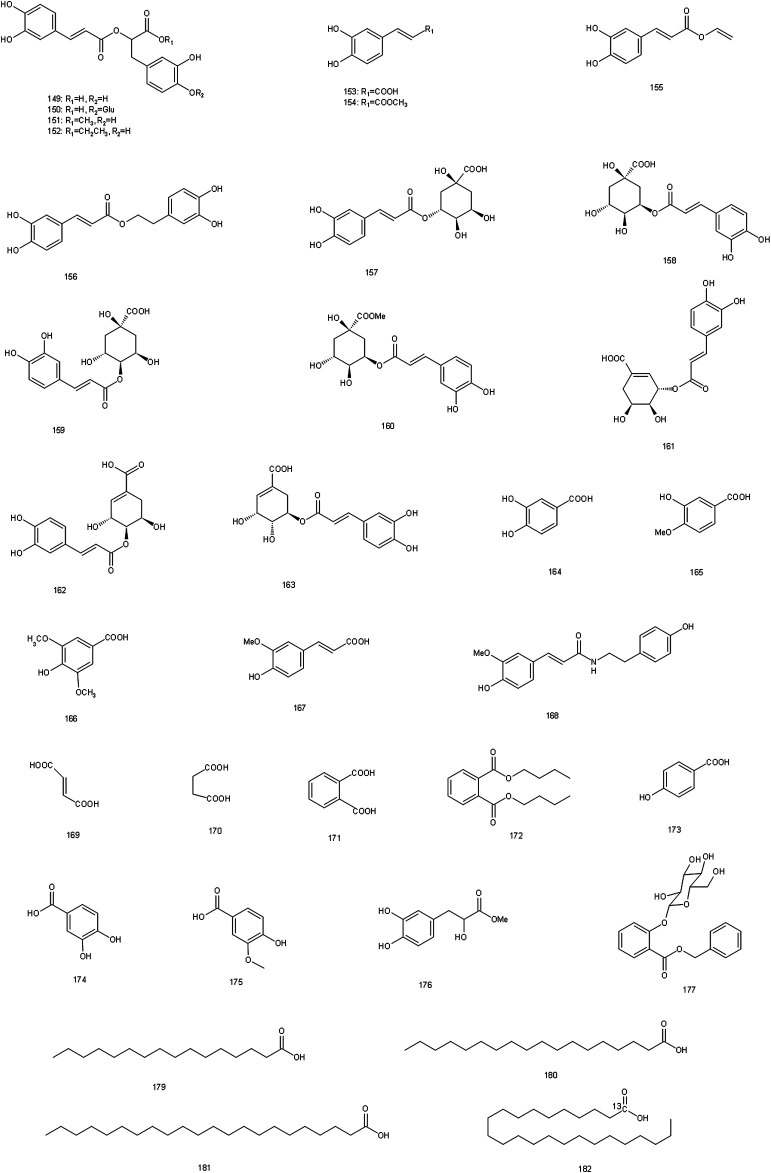
Chemical structures of organic acids identified in S. glabra extract.

**FIGURE 6:**
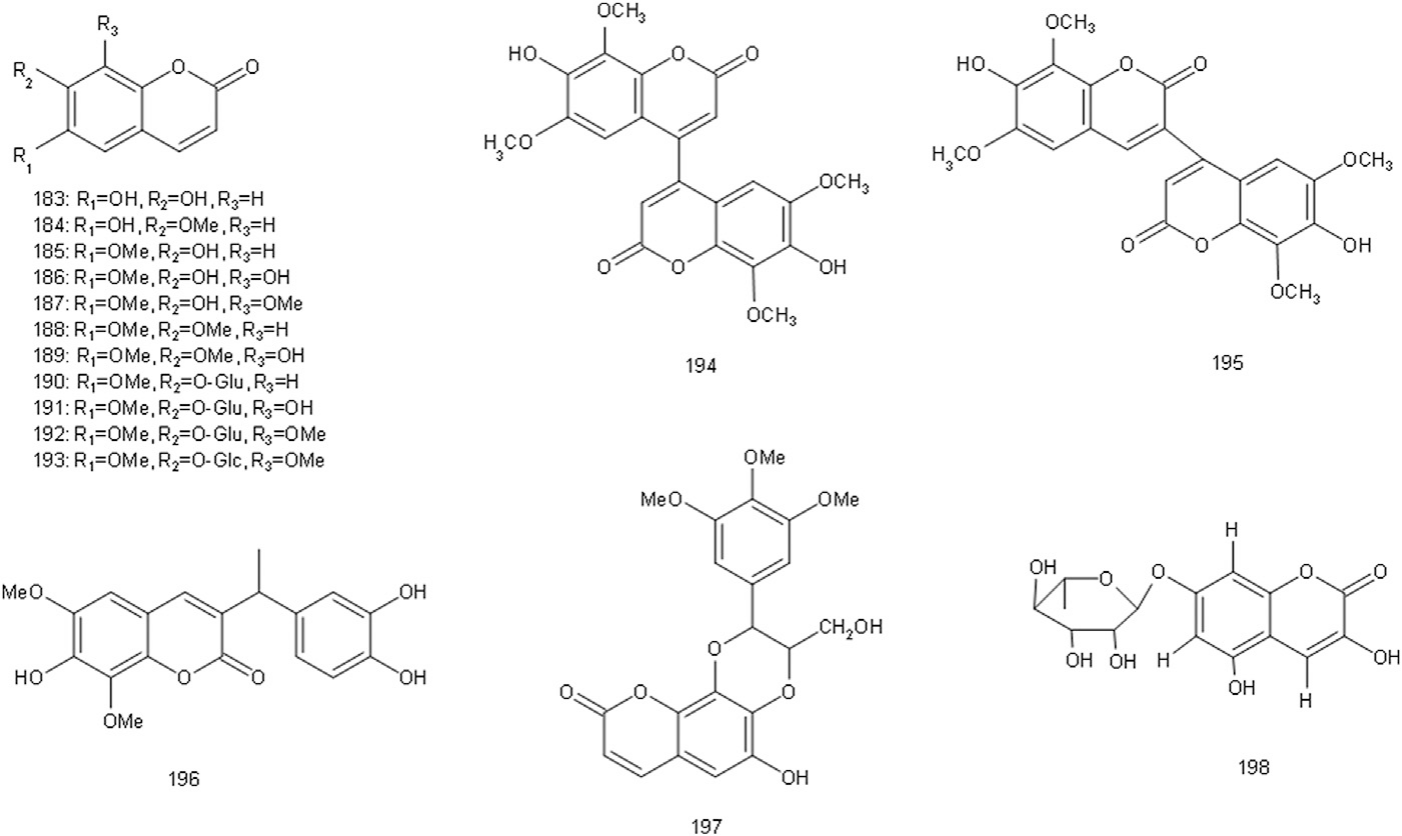
Chemical structures of coumarins identified in S. glabra extract.

**FIGURE 7:**
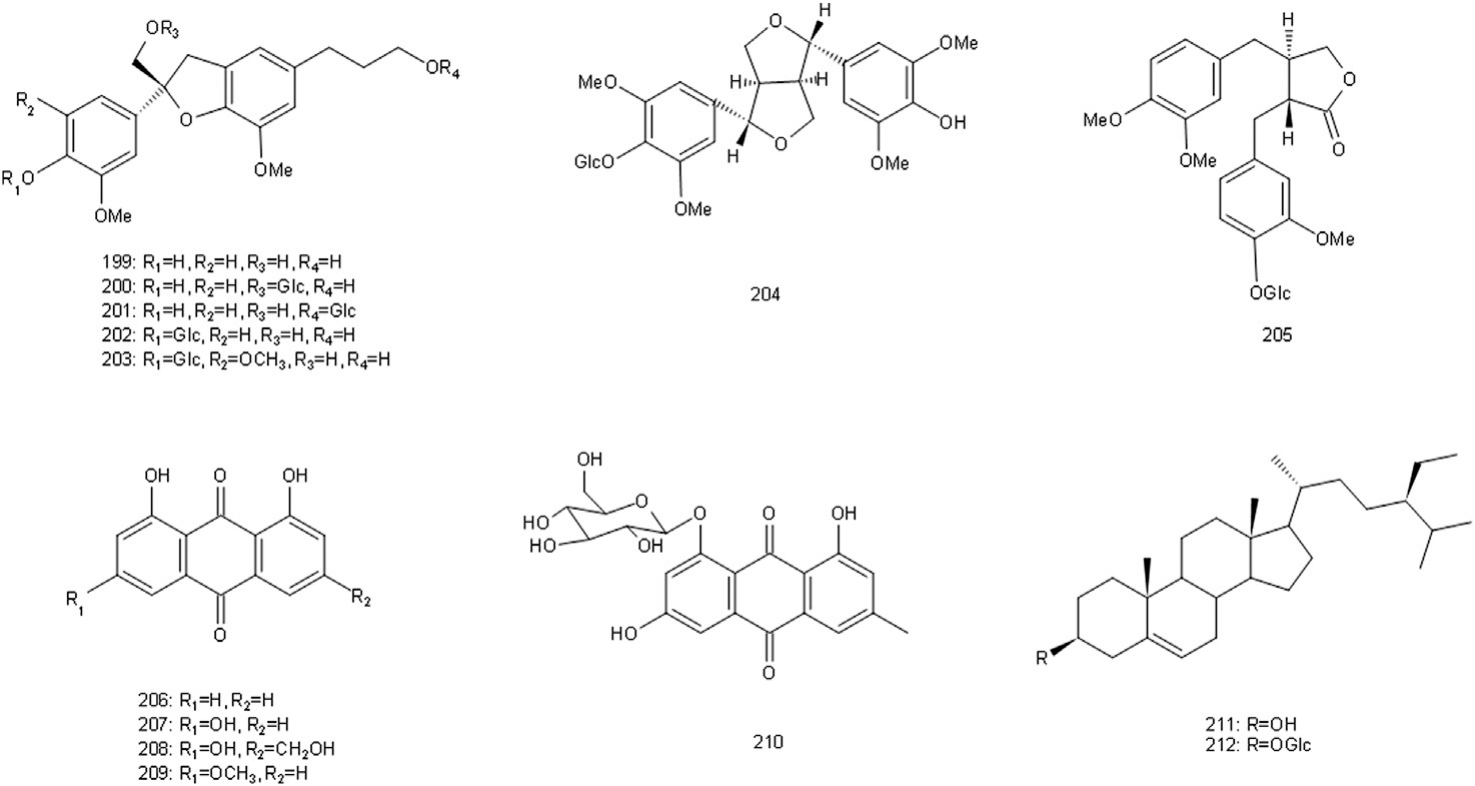
Chemical structures of lignins, anthraquinones and steroids identified in S. glabra extract.

Since the 1970s, the chemical constituents of *S. glabra* have gained the interest of the scholars at home and abroad. Up to now, over 200 compounds have been isolated and identified from *S. glabra*, including sesquiterpenes, flavonoids, phenolic acids, coumarins, lignans, anthraquinones, volatile oil, a small quantity of amino acids, trace elements, polysaccharides and proteoglycans. Among them, flavonoids are considered to be the main active components in *S. glabra*. The chemical constituents reported are listed in Table 1 and their corresponding structures are shown in Figures 3–7.

**TABLE 1:** Compounds presenting in *S. glabra*.

No	Chemical component	Extract	Part	References
	Sesquiterpenes			
1	Chloranthalactone A	Dichloromethane	Aerial parts	Tsui and Brown (1996)
2	Chloranthalactone B	EtOH	Whole plant	Hu et al. (2009)
3	Chloranthalactone E	EtOH	Whole plant	Zhu et al. (2008b)
4	Chloranthalactone E 8-O-β-D-glucopyranoside	EtOH	Whole plant	Li et al. (2006a)
5	Chloranthalactone F	Et_2_O	Leaves	Takeda et al. (1993)
6	Chloranthalactone G	Dichloromethane	Aerial parts	Tsui and Brown, (1996)
7	Chloranoside A	EtOH	Whole plant	Hu et al. (2009)
8	Chloranoside B	Et_2_O	Leaves	Takeda et al. (1993)
9	Chloranthalactone A photodimer	Acetone	Leaves	Okamura et al. (1995)
10	Sarcandralactone A	EtOH	Whole plant	He et al. (2010)
11	Sarcandralactone B	EtOH	Whole plant	He et al. (2010)
12	Sarcandralactone C	EtOH	Whole plant	Ni et al. (2013)
13	Sarcandralactone D	EtOH	Whole plant	Ni et al. (2013)
14	Sarcandralactone E	EtOH	Whole plant	Ni et al. (2013)
15	8β, 9α-dihydroxylindan-4(5),7(11) -dien-8α,12-olide	EtOH	Whole plant	Zhu et al. (2008b)
16	9-hydroxyheterogorgiolide	EtOH	Whole plant	Hu et al. (2009)
17	Shizukanolide E	EtOH	Whole plant	Hu et al. (2013)
18	Shizukanolide F	EtOH	Whole plant	Hu et al. (2013)
19	Shizukanolide H	EtOAc	Whole plant	Zheng et al. (2014)
20	4α-hydroxy-5α*H*-lindan-8 (9)-en-8,12-olide	EtOH	Whole plant	Li et al. (2011)
21	Chlorajapolide C	EtOAc	Whole plant	Zheng et al. (2014)
22	Sarcaglabrin A	MeOH	Aerial parts	Yang et al. (2020)
23	Glabranol A	EtOH	Aerial parts	Oanh et al. (2010)
24	Glabranol B	EtOH	Aerial parts	Oanh et al. (2010)
25	Sarcaglaboside A	EtOH	Whole plant	Li et al. (2006a)
26	Sarcaglaboside B	EtOH	Whole plant	Li et al. (2006a)
27	Sarcaglaboside C	EtOH	Whole plant	Li et al. (2006a)
28	Sarcaglaboside D	EtOH	Whole plant	Li et al. (2006a)
29	Sarcaglaboside E	EtOH	Whole plant	Li et al. (2006a)
30	Sarcaglaboside F	EtOH	Whole plant	Hu et al. (2009)
31	Sarcaglaboside G	EtOH	Whole plant	Hu et al. (2009)
32	Sarcaglaboside H	EtOH	Whole plant	Hu et al. (2009)
33	Atractylenolide II	Et_2_O	Leaves	Takeda et al. (1993)
34	Atractylenolide III	EtOH	Whole plant	Wang et al. (2007)
35	Atractylenolide IV	EtOH	Whole plant	Hu et al. (2013)
36	8β,9α-dihydroxyeudesman-4(15),7(11)-dien-8α,12-olide	EtOH	Whole plant	Zhu et al. (2008b)
37	Neolitacumone B	EtOH	Whole plant	Ni et al. (2013)
38	1α,8α,9α-trihydroxyeudesman-3(4),7(11)-dien-8β,12-olide	EtOH	Whole plant	Wang et al. (2012)
39	3-eudesmene-1β,7, 11-triol	EtOH	Whole plant	He et al. (2010)
40	(-)-istanbulin A	EtOH	Whole plant	Zhu et al. (2008b)
41	Istanbulin A	EtOAc	Whole plant	Zheng et al. (2014)
42	Istanbulin B	EtOAc	Whole plant	Zheng et al. (2014)
43	Furanodienone	EtOH	Whole plant	Luo et al. (2005a)
44	(-)-4β,7α-Dihydromadendrane	Et_2_O	Leaves	Takeda et al. (1993)
45	Spathulenol	Dichloromethane	Aerial parts	Tsui and Brown, (1996)
46	PipelolA	EtOH	Whole plant	Wang et al. (2010a)
47	Sarcaboside A	EtOH	Whole plant	Li et al. (2012a)
48	Sarcaboside B	EtOH	Whole plant	Li et al. (2012a)
49	Glabralide A	EtOH	Whole plant	Yang et al. (2018)
50	Glabralide B	EtOH	Whole plant	Yang et al. (2018)
51	Glabralide C	EtOH	Whole plant	Yang et al. (2018)
52	Sarcandrolide A	EtOH	Whole plant	He et al. (2010)
53	Sarcandrolide B	EtOH	Whole plant	He et al. (2010)
54	Sarcandrolide C	EtOH	Whole plant	He et al. (2010)
55	Sarcandrolide D	EtOH	Whole plant	He et al. (2010)
56	Sarcandrolide E	EtOH	Whole plant	He et al. (2010)
57	Sarcandrolide F	EtOH	Whole plant	Ni et al. (2013)
58	Sarcandrolide G	EtOH	Whole plant	Ni et al. (2013)
59	Sarcandrolide H	EtOH	Whole plant	Ni et al. (2013)
60	Sarcandrolide I	EtOH	Whole plant	Ni et al. (2013)
61	Sarcandrolide J	EtOH	Whole plant	Ni et al. (2013)
62	Sarcaglabrin B	MeOH	Aerial parts	Yang et al. (2020)
63	Sarcaglabrin C	MeOH	Aerial parts	Yang et al. (2020)
64	Shizukaol A	EtOH	Roots	Wei et al. (2019)
65	Shizukaol B	EtOH	Seeds	Wang et al. (2015b)
66	Shizukaol C	EtOH	Seeds	Wang et al. (2015b)
67	Shizukaol D	EtOH	Whole plant	Ni et al. (2013)
68	Shizukaol E	EtOH	Roots	Wei et al. (2019)
69	Shizukaol G	EtOH	Seeds	Wang et al. (2015b)
70	Shizukaol H	EtOH	Whole plant	Ni et al. (2013)
71	Shizukaol I	EtOH	Whole plant	Luo. (2004)
72	Shizukaol N	EtOH	Seeds	Wang et al. (2015b)
73	Sarglabolide A	EtOH	Seeds	Wang et al. (2015b)
74	Sarglabolide B	EtOH	Seeds	Wang et al. (2015b)
75	Sarglabolide C	EtOH	Seeds	Wang et al. (2015b)
76	Sarglabolide D	EtOH	Seeds	Wang et al. (2015b)
77	Sarglabolide E	EtOH	Seeds	Wang et al. (2015b)
78	Sarglabolide F	EtOH	Seeds	Wang et al. (2015b)
79	Sarglabolide G	EtOH	Seeds	Wang et al. (2015b)
80	Sarglabolide H	EtOH	Seeds	Wang et al. (2015b)
81	Sarglabolide I	EtOH	Seeds	Wang et al. (2015b)
82	Sarglabolide J	EtOH	Seeds	Wang et al. (2015b)
83	Sarglabolide K	EtOH	Seeds	Wang et al. (2015b)
84	Chlorajaponilide E	EtOH	Whole plant	Ni et al. (2013)
85	Chlorahololide F	EtOH	Whole plant	Ni et al. (2013)
86	Spicachlorantin F	EtOH	Whole plant	Ni et al. (2013)
87	Chlorahololide D	EtOH	Roots	Wei et al. (2019)
88	Henriol D	EtOH	Whole plant	Ni et al. (2013)
89	Cycloshizukaol A	EtOH	Roots	Wei et al. (2019)
90	Sarglaperoxide A	EtOH	Seeds	Wang et al. (2016)
91	Sarglaperoxide B	EtOH	Seeds	Wang et al. (2016)
92	Dihydrovomifoliol	Acetone	Whole plant	Wu et al. (2012b)
93	Dihydrovomifoliol-O-β-D-glucopyranoside	Acetone	Whole plant	Wu et al. (2012b)
94	Drovomifoliol-O-β-D-glucopyranoside	Acetone	Whole plant	Wu et al. (2012b)
95	Cis-abscisic acid	Acetone	Whole plant	Wu et al. (2012b)
96	β-D-glucopyranosylabscizate	Acetone	Whole plant	Wu et al. (2012b)
97	Asicariside B1	Acetone	Whole plant	Wu et al. (2012b)
	Diterpenes			
98	15-hydroxy-12-oxolabda-8-(17),13E-dien-19-oicacid	EtOH	Whole plant	Luo. (2004)
99	12R,15-dihydroxylabda-8 (17),13E-dien-19-oicacid	EtOH	Whole plant	Luo. (2004)
100	12S,15-dihydroxylabda-8 (17),13E-dien-19-oicacid	EtOH	Whole plant	Luo. (2004)
101	9R-12S,15-dihydroxylabda-8 (17),13E-dien-19-oic acid	EtOH	Whole plant	Luo. (2004)
	Triterpenes			
102	Sarcandroside A	MeOH	Whole plant	Luo et al. (2005b)
103	Sarcandroside B	MeOH	Whole plant	Luo et al. (2005b)
104	Lupeol	EtOH	Whole plant	Luo et al. (2005a)
105	24-hydroxy lupeol	EtOH	Whole plant	Luo et al. (2005a)
106	Betulinic acid	Dichloromethane	Aerial parts	Tsui and Brown, (1996)
107	Ursolic acid	EtOH	Whole plant	Fu and Liang, (2013)
108	Oleanolic acid	EtOH	Whole plant	Fu and Liang, (2013)
	Flavonoids			
109	Kaempferol	Aqueous	Whole plant	Yuan et al. (2008)
110	Kaempferol-3-O-β-D-glucuronide	Aqueous	Whole plant	Huang et al. (2008)
111	Quercetin	EtOH	Whole plant	Zou et al. (2007)
112	Quercetin-3-O-glucuronide	Aqueous	Stems	Duan et al. (2010)
113	Quercetin-3-O-β-D-glucuronopyranoside methyl ester	Aqueous	Whole plant	Huang et al. (2008)
114	Quercetin-3-O-α-D-glucuronide	Aqueous	Whole plant	Huang et al. (2008)
115	Quercetin-3-O-α-L-rhamnoside	EtOH	Whole plant	Tong et al. (2010)
116	Rutin	EtOH	Whole plant	Fu and Liang, (2013)
117	Hyperoside	EtOH	Whole plant	Fu and Liang, (2013)
118	Epimedin C	Aqueous	Whole plant	Li et al. (2010)
119	Astilbin	EtOH	Whole plant	Wang et al. (2010b)
120	Neoastilbin	EtOH	Whole plant	Wang et al. (2010b)
121	Isoastilbin	EtOH	Whole plant	Wang et al. (2010b)
122	Neoisoastilbin	EtOH	Whole plant	Wang et al. (2010b)
123	Pinostrobin	EtOH	Whole plant	Wang et al. (2007)
124	7-Methylnaringenin	EtOH	Whole plant	Luo et al. (2005a)
125	5-hydroxy-7-methoxy-dihyflavanones	EtOH	Whole plant	Luo et al. (2005a)
126	5-hydroxy-7, 4′-dimethoxyflavanone	EtOH	Whole plant	Wang et al. (2007)
127	(+)-3,3′,5,5′,7-pentahydroxy-diflavanone	EtOH	Whole plant	Zhu et al. (2008a)
128	5-dihydroxy-7,4′-dimethoxy-dihyflavanones	Dichloromethane	Aerial parts	Tsui and Brown, (1996)
129	5,4′-dihydroxy-7-methoxy-dihyflavanones	EtOH	Whole plant	Luo et al. (2005a)
130	5,7,4′-trihydroxy-8-C-β-D-glucopyranosylflavanone	Aqueous	Whole plant	Huang et al. (2008)
131	5,7,3′,4′-tetrahydroxy-6-C-β-D-glucopyranosylflavanone	Aqueous	Whole plant	Yuan et al. (2008)
132	Isoliquiritigenin	EtOH	Whole plant	Zou et al. (2007)
133	2′,4′-dihydroxy-6′-methoxy-dihydrochalcone	Dichloromethane	Aerial parts	Tsui and Brown, (1996)
134	2′,4′-dihydroxy-4,6′-dimethoxy-dihydrochalcone	Dichloromethane	Aerial parts	Tsui and Brown, (1996)
135	2′,6′-dihydroxy-4′-methoxydihydrochalcone	Dichloromethane	Aerial parts	Tsui and Brown, (1996)
136	2′,6′-dihydroxy-4,4′-dimethoxy-dihydrochalcone; calomelanen	Dichloromethane	Aerial parts	Tsui and Brown, (1996)
137	2′-hydroxy-4′,6′-dimethoxy-dihydrochalcone	Dichloromethane	Aerial parts	Tsui and Brown, (1996)
138	2′-hydroxy-4,4′,6′-timethoxy-dihydrochalcone	Dichloromethane	Aerial parts	Tsui and Brown, (1996)
139	3'-(7″-allylphenyl) -2′,4′,4′-trihydroxy-6′- methoxydihydrochalcone	EtOH	Whole plant	Li et al. (2006b)
140	Cilicicone B	MeOH	Whole plant	Zheng et al. (2016)
141	β,2,3′,4,4′,6-Hexahydroxy-α-(α-L-rhamnopyranosyl) dihydrochalcone	MeOH	Whole plant	Zheng et al. (2016)
142	Catechin 3-O-α-l-rhamnopyranoside	EtOH	Whole plant	Li. (2006)
143	Pelargonidin 3-rhamnosylglucoside	/	Fruits	Ishikura. (1971)
144	Cyaniding 3-rhamnosylglucoside	/	Fruits	Ishikura. (1971)
145	Glabraoside A	EtOH	Whole plant	Li et al. (2006b)
146	Glabraoside B	EtOH	Whole plant	Li. (2006)
147	Glabraoside C	EtOH	Whole plant	Wang et al. (2012)
148	Glabraoside D	EtOH	Whole plant	Wang et al. (2012)
	Organic acids			
149	Rosmarinic acid	Aqueous	Whole plant	Huang et al. (2007)
150	Rosmarinic acid-4-O-β-D-glucoside	Aqueous	Whole plant	Li et al. (2010)
151	Methyl rosmarinate	Aqueous	Whole plant	Huang et al. (2007)
152	Ethyl rosmarinate	EtOH	Whole plant	Zhu et al. (2008a)
153	Caffeic acid	Aqueous	Whole plant	Huang et al. (2007)
154	Caffeic acid ethyl ester	EtOH	Whole plant	Li et al. (2012b)
155	Vinyl caffeate	EtOH	Whole plant	Li et al. (2012b)
156	Caffeic acid 3,4-dihydroxyphenethyl ester	EtOH	Whole plant	Lian. (2006)
157	Chlorogenic acid	EtOH	Whole plant	Li et al. (2012b)
158	Neochlorogenic acid	EtOH	Whole plant	Li et al. (2012b)
159	Cryptochlorogenic acid	EtOH	Whole plant	Li et al. (2012b)
160	Methyl 5-O-caffeoylquinilic acid	Aqueous	Whole plant	Huang et al. (2008)
161	3-O-caffeoylshikimic acid	EtOH	Whole plant	Li et al. (2012b)
162	4-O-caffeoylshikimic acid	EtOH	Whole plant	Li et al. (2012b)
163	5-O-caffeoylshikimic acid	EtOH	Whole plant	Li et al. (2012b)
164	Protocatechuic acid	EtOH	Whole plant	Li et al. (2012b)
165	Isovanillic acid	Aqueous	Stems	Duan et al. (2010)
166	Caryophylic acid	Aqueous	Whole plant	Li et al. (2010)
167	Ferulic acid	EtOH	Whole plant	Li. (2006)
168	N-trans-feruloyltyramine	EtOH	Whole plant	Zhu et al. (2008a)
169	Fumarc acid	Aqueous	Whole plant	Wang and Ma, (1979a)
170	Succinic acid	EtOH	Whole plant	Tong et al. (2010)
171	Phthalic acid	EtOH	Whole plant	Tong et al. (2010)
172	Dibutyl phthalate	Aqueous	Whole plant	Huang et al. (2007)
173	P-hydroxybenzoic acid	Aqueous	Whole plant	Li et al. (2010)
174	3,4-dihydroxy benzoic acid	Aqueous	Whole plant	Huang et al. (2007)
175	3-methoxy-4-hydroxybenzoic acid	Aqueous	Whole plant	Li et al. (2010)
176	Methyl 3,4-dihydroxyphenyll actate	Aqueous	Whole plant	Huang et al. (2007)
177	Benzyl 2-β-glucopyranosyloxybenzoate	Acetone	Whole plant	Wu et al. (2012a)
178	Palmitic acid	EtOH	Whole plant	Wang et al. (2007)
179	Stearic acid	EtOH	Whole plant	Zeng and Luo, (2005)
180	N-pentadecanoic acid	EtOH	Whole plant	Wang et al. (2007)
181	N-docosanoic acid	EtOH	Whole plant	Tong et al. (2010)
182	N-heptadecanoic acid	EtOH	Whole plant	Tong et al. (2010)
	Coumarins			
183	Esculetin	EtOH	Whole plant	Xu et al. (2008)
184	Isoscopoletin	EtOH	Whole plant	Wang et al. (2010b)
185	Scopletin	EtOH	Whole plant	Xu et al. (2008)
186	Fraxetin	EtOH	Whole plant	Xu et al. (2008)
187	Isofraxidin	EtOH	Whole plant	Wang et al. (2007)
188	Scoparone	EtOH	Whole plant	Wang et al. (2007)
189	Fraxidin	Aqueous	Whole plant	Yuan et al. (2008)
190	Scopolin	Acetone	Whole plant	Wu et al. (2012b)
191	Fraxin	EtOH	Whole plant	Xu et al. (2008)
192	Isofraxidin-7-O-β-D-glucopyranoside	Aqueous	Whole plant	Yuan et al. (2008)
193	Eleutheroside B1	EtOH	Whole plant	Luo et al. (2005a)
194	3,3′-biisofraxidin	EtOH	Whole plant	Wang et al. (2007)
195	4,4′-bisofraxidin	EtOH	Whole plant	Xu et al. (2008)
196	Sarcandracourmarin	Aqueous	Whole plant	Feng et al. (2010)
197	Hemidesmin 1	EtOH	Whole plant	Zhu et al. (2008a)
198	3,5-dihydroxycoumarin-7-O-α-L-rhamnopyranoside	EtOH	Whole plant	Wang et al. (2015a)
	Lignans			
199	(-)-(7S,8R)-dihydrodehydrodiconiferyl alcohol	EtOH	Whole plant	Zhu et al. (2008a)
200	(-)-(7S,8R)-dihydrodehydrodiconiferyl alcohol-9-O-α-D-glucopyranoside	Acetone	Whole plant	Wu et al. (2012a)
201	(-)-(7S,8R)-dihydrodehydrodiconiferyl alcohol-9′-O-α-D-glucopyranoside	Acetone	Whole plant	Wu et al. (2012a)
202	(-)-(7S,8R)-dihydrodehydrodiconiferyl alcohol-4-O-α-D -glucopyranoside	Acetone	Whole plant	Wu et al. (2012a)
203	(-)-(7S,8R)-5-Methoxydihydrodehydrodiconiferyl alcohol-4-O-β-D-glucopyranoside	Acetone	Whole plant	Wu et al. (2012a)
204	Syringaresinol monoside	EtOH	Whole plant	Wang et al. (2010b)
205	Styraxiaponoside B	EtOH	Whole plant	Wang et al. (2010b)
	Anthraquinones			
206	Chrysophanol	EtOH	Whole plant	Fu and Liang, (2013)
207	Emodin	EtOH	Whole plant	Yu et al. (2012)
208	Citreorosein	EtOH	Whole plant	Fu and Liang, (2013)
209	Physcion	EtOH	Whole plant	Yu et al. (2012)
210	Emodin-8-O-β-d-glucopyranoside	EtOH	Whole plant	Fu and Liang, (2013)
	Steroids			
211	β-sitosterol	EtOH	Whole plant	Wang et al. (2007)
212	Daucosterol	EtOH	Whole plant	Wang et al. (2007)

### Terpenoids

There are sesquiterpenes (**1–97**), diterpenes (**98–101**) and triterpenes (**102–108**) in *S. glabra*, among them, sesquiterpenes are the most abundant substances, including the characteristic components such as chloranthalactone, chloranoside, sarcandralactone, shizukaol, and sarglabolide. Sesquiterpenes isolated and identified from *S. glabra* have been reported to possess anti-inflammatory, antibacterial and antitumor effects, *etc*. (He et al., 2010; Wang P. et al., 2015, Wang et al., 2016). For instance, chloranthalactone E (**3**), atractylenolide III (**34**) and sarcandrolides A-C (**52–54**) exhibited reportedly antitumor effects (Wang et al., 2007; He et al., 2010), while shizukaol B (**65**), shizukaol G (**69**) and sarglabolide A (**73**) showed anti-inflammatory activities (Wang P. et al., 2015). Sarglaperoxide A (**90**) possessed anti-inflammatory and antibacterial effects, inhibiting 53.6% nitric oxide (NO) production at 25 μM and 64.5% *Staphylococcus aureus* growth at 25 μg/ml (Wang et al., 2016).

### Flavonoids

So far, over 40 flavonoids have been found in *S. glabra* (**109–148**). Flavonoids are the main components within *S. glabra*, and now are considered to be the main bioactive components in the treatment of thrombocytopenia. Flavonoids are also often used as important indicators to control the quality of *S. glabra*. Astilbin (**119**), as one of the active components of *S. glabra*, was reported to play an anti-thrombocytopenic role in rat bone marrow megakaryocytes by up-regulating transforming growth factor beta (TGF-β1) content and down-regulating thermoplastic polyolefin (TPO) content, which may be the effective component against thrombocytopenia (Tang et al., 2014). Besides, there were differences in the content of total flavonoids in different parts of *S. glabra*. The content of total flavonoids in leaves reached 3.17%, which was higher than that in roots (2.38%) and stems (2.11%) (Li et al., 2007). The results suggested that the medicinal part could be selected according to the clinical needs, which was beneficial to the sustainable utilization of *S. glabra*.

### Organic Acids

At present, more than 20 organic acids have been isolated from *S. glabra* (**149–182**), which can be divided into phenolic acids and fatty acids. Phenolic acids are important components in *S. glabra*, containing rosmarinic acid (**149**), caffeic acid (**153**), chlorogenic acid (**157**), neochlorogenic acid (**158**), cryptochlorogenic acid (**159**), and other components with significant pharmacological activities. They might be the bioactive components of *S. glabra* to exert antibacterial, anti-inflammatory, and antioxidant effects, *etc*. Among them, rosmarinic acid (**149**) possessed various pharmacological effects including anti-inflammatory, antibacterial, antiviral, antioxidant, and anti-tumor effects, its anti-inflammatory and antioxidant effects were particularly significant (Nunes et al., 2017). Rosmarinic acid is also one of the phenolic acids with the highest content within *S. glabra*, serving as a marker in *Chinese pharmacopoeia* for controlling the quality of *S. glabra*.

### Coumarins

Currently, more than a dozen coumarins have been isolated from *S. glabra* (**183–198**). As the most representative coumarin with strong pharmacological activity, isofraxidin (**187**) is used as an index for controlling the quality of *S. glabra* and its preparations by *Chinese Pharmacopoeia*. Studies have shown that isofraxidin has a wide range of pharmacological effects (Li et al., 2014; Liu et al., 2015; Jin et al., 2020), including anti-inflammatory, anti-viral, and anti-tumor effects, as well as inhibition of platelet aggregation*.* Furthermore, 3,3′-biisofraxidin (**194**) had been reported to induce gastric cancer cells apoptosis by activating the mitochondrial-mediated apoptosis pathway (Wu et al., 2015).

### Other Compounds

There are lignans (**199–205**), anthraquinones (**206–210**) and steroids (**211–212**) in *S. glabra*. Furthermore, there are abundant volatile components in *S. glabra* (Yang R. et al., 2008), mainly including α-pinene, β-phellandrene, and α-thujene. It also contains 16 kinds of amino acids, such as aspartic acid, glutamic acid, leucine and so on, six kinds of which are essential amino acids for human body, as well as trace elements including iron, zinc, calcium, magnesium and so on (Yang B. et al., 2008). In addition, acidic polysaccharide and proteoglycan are also isolated from *S. glabra* (Liu W. et al., 2017; Sun et al., 2020).

## Pharmacology

Pharmacological studies have indicated that *S. glabra* has a wide range of pharmacological effects, including antibacterial, antiviral, anti-inflammatory, anti-tumor, anti-oxidant, anti-thrombocytopenic effects, *etc*. Pharmacological effects of *S. glabra* and its preparations as well as monomeric compounds were summarized in Table 2, which were described in the following sections as well.

**TABLE 2:** Modern Pharmacological studies of *S. glabra*.

Effect	Model	Part of plant/Extracts or compound	Positive control	Formulation/dosage	Result/mechanism	References
Antibacterial	*Streptococcus mutans*	Ethanol	/	*in vitro*: 3.125, 6.25, 12.5, 25, 50, 100 mg/ml	Inhibiting the bacterial growth and its glucosyltransferase activity	Huang and He. (2001)
	*Helicobacter pylori* and its drug-resistant bacteria	The whole plant/Aqueous	/	*in vitro*: 95 μg/ml	Damaging the function of outer membrane barrier	Guo. (2015)
	*Streptococcus mutans*	Compound **110**	/	*in vitro*: 1.0 mg/ml	Its diameter of bacteriostatic circle was 14.67 ± 0.08 mm	Yuan et al. (2008)
Antiviral	Mice infected with H_1_N_1_ virus	Ethanol	Ribavirin reduced oxidative stress levels to alleviate lung injury in mice	*in vivo*: 75 mg/kg	Activating Nrf2/HO-1 pathway to regulate SOD, MDA, and NO.	Huo et al. (2020)
	Mice infected with A/FM/1/47 H_1_N_1_ virus	Compound **150**	The high-dose group reduced viral replication in the lungs, and its effect was similar to that of ribavirin (50 mg/kg)	*in vivo*: 20, 50 mg/kg	Reducing pulmonary edema, inflammatory reaction, oxidative damage and viral replication in the lungs	Liu et al. (2017a)
	RNP virus	Compound **193**	/	*in vitro*: 50 μg/ml	Reducing RN mRNA expression	Wang et al. (2017)
Anti-inflammatory	LPS-induced RAW264.7 macrophage	The whole plant/Ethyl acetate extract and polysaccharide	/	*in vitro*: 100, 200 μg/ml	Inhibiting RAW264.7 cells proliferation and NO expression	Xie et al. (2010)
	LPS-induced RAW264.7 macrophage	Compound **67**	/	*in vitro*: 5, 10, 15, 20 μM	Activating akt mediated Nrf2/HO-1 pathway and inhibiting NF-κB activation	Wei et al. (2019)
	LPS-induced inflammatory mice	Compound **187**	/	*in vivo*: 1, 5, 15 mg/kg	Down-regulating TNF-α expression by inhibiting NF-κB pathway	Liu et al. (2015)
Anti-tumor	Lung cancer cells A-549, colon cancer cells HCT-29, gastric cancer cells BGC-823	*Zhongjiefeng* injection	/	*in vitro*: 3.125, 6.25, 12.5 25, 50 μg/ml	The IC_50_ values of A-549, HCT-29 and BGC-823 cells were 15.18, 29.21 and 38.58 μg/ml respectively	Zhao et al. (2008)
	Non-small cell lung cancer A549 and H1299	*Zhongjiefeng* tablets	/	*in vitro*: 0.625, 1, 1.25 mg/ml	Up-regulating the TGF-β pathway to induce P21 expression, blocking the cancer cell cycle in the G0/G1 phase	Chen et al. (2018)
	Leukemia cells K562	The whole plant/Total flavonoids	/	*in vitro*: 25, 50, 100 μg/ml	Down-regulating Bcl-2, Caspase-3 protein expression and up-regulating cleaved Caspase-3 protein expression	Sun et al. (2019)
	Osteosarcoma cells MG-63	The whole plant/Polysaccharide	/	*in vitro*: 31.25, 62.5, 125 nM	Down-regulating the ERK/eIF4F/Bcl-XL pathway to promote the release of cytochrome C and activate the caspase protein	Zhang et al. (2014)
	S-180 cell-derived tumor model mice	The whole plant/Polysaccharide	/	*in vivo*: 25, 50, 100 mg/kg	Inhibition of transplanted tumor growth	Zhang et al. (2014)
Immune regulation	RAW264.7 macrophage cells	The whole plant/Polysaccharide	/	*in vitro*: 25, 50, 100 mg/l	Increasing CD40, CD14 expression, as well as IL-1β, TNF-α, iNOS and IL-10 mRNA expression, and decreasing CD16/32 expression	Jiang et al. (2014)
	Restrained stress mice	The whole plant/Aqueous	/	*in vivo*: 125 mg/kg	Improving immune cells proportion and number	He et al. (2009a)
	Restrained stress mice	The whole plant/Aqueous	/	*in vivo*: 125 mg/kg	Partly through improving the ability of antioxidant to enhance immunity	He et al. (2009b)
Antioxidant	Hydroxy radical	The whole plant/Aqueous	/	*in vitro*: 0.2, 0.4, 0.6, 1.2 mg/ml	At the concentration of 1.2 mg/ml, its scavenging rate reached 89.89%	Qin et al. (2007)
	DPPH radical	The whole plant/Aqueous	Quercetin and lutin half scavenging concentrations were 4.39 mg/L and 7.52 mg/L respectively	*in vitro*: 1, 3, 10, 30, 100 mg/l	Its half scavenging concentration was 13.49 mg/l	Li et al. (2009)
	Hydroxy, superoxide anion, DPPH, and ABTS radicals, and Fe^2+^	The whole plant/Polysaccharide	Ascorbic acid (0.5–2.0 mg/ml) showed significant free radical scavenging activity	*in vitro*: 0.5, 1.0, 1.5, 2.0 mg/ml	Scavenging these free radicals effectively and chelating Fe^2+^	Jin et al. (2012)
	Mesenchymal stem cells	The whole plant/ethanol, compound **119** and **149**	/	*in vitro*: 10–100 μg/ml, and 20–110 μg/ml	Protecting mesenchymal stem cells from oxidative stress and hydroxy radical mediated DNA damage	Liu et al. (2016)
Anti-thrombocytopenic	Bone marrow stromal cell-Megakaryocyte co-culture system	The whole plant/Total flavonoids	/	*in vitro*: 1.95, 3.90, 7.80 μg/ml	Increasing the content of TPO, SDF-1 and VCAM-1, and decreasing the content of TGF-β1	Lu et al. (2019)
	Cytarabine-induced thrombocytopenia mice	The whole plant/Total flavonoids	The activity of prednisolone acetate (10 mg/kg) in promoting TPO and C-mpl expression was weaker than the extract	*in vivo*: 31.5, 63.0, 94.5 mg/kg	Promoting the expression of TPO and its receptor C-mpl	Lu et al. (2018a)
	Cytarabine-induced thrombocytopenia mice	The whole plant/Total flavonoids	The activity of prednisolone acetate (10 mg/kg) in promoting SDF-1 and CXCR-4 expression was weaker than the extract	*in vivo*: 31.5, 63.0, 94.5 mg/kg	Promoting SDF-1 and its receptor CXCR-4 expression	Lu et al. (2018b)
Hepatoprotec-tive	Dimethylnitrosamine-indued liver injury rat	*Zhongjiefeng* tablets	/	*in vivo*:/	Normalizing the serum protein index, and improving the level of antioxidant index	Jin and Li. (1998)
	*p*.acnes-LPS-induced immunological hepatitis mice	Extract	The inhibitory effect on ALT activity of cyclosporin a (1 mg/kg) was 85.84%	*in vivo*: 125 mg/kg	Inhibiting ALT activity, and the inhibition rate reached 78.5%	Li et al. (2008)
Hypoglycemic	α-glucosidase	The whole plant/Polysaccharide	Acarbose (15.63–250 μg/ml) inhibited α-glucosidase activity with a IC_50_ value of 148.3 μg/ml	*in vitro*: 15.63–250 μg/ml	The inhibitory effect of polysaccharide on α-glucosidase (IC_50_ = 49.01 μg/ml) was stronger than that of positive control	Liu et al. (2017b)
	HFD and STZ-induced diabetic mice	The whole plant/Polysaccharide	Polysaccharide was superior to acarbose (10 mg/kg) and metformin (200 mg/kg) in reducing fasting blood glucose levels and relieve the insulin resistance	*in vivo*: 100, 200 mg/kg	Reducing insulin resistance, improving lipid metabolism, increasing glucose utilization and antioxidant capacity	Liu et al. (2017b)
Hypolipidemic	HFD-induced hyperlipidemic mice	The whole plant/Total flavonoids	The hypolipidemic effect of the high-dose group was equivalent to that of lovastatin (4.0 mg/d)	*in vivo*: 1.0, 2.0, 4.0 mg/d	Decreasing triglyceride, total cholesterol, and low density lipoprotein	Ji. (2012)

### Antibacterial

Studies had shown that *S. glabra* possessed a broad spectrum of antibacterial effects, which had inhibitory effects on *Staphylococcus aureus* and its drug resistant bacteria, *Pseudomonas eruginosa*, *Escherichia coli*, *Streptococcus pneumoniae*, *Dysentery bacilli*, *Typhoid* and *Paratyphoid bacilli*, especially on *S. aureus* and *P. aeruginosa*, it showed strong antibacterial activity (Jiang et al., 2000; Wang and Du, 2008). *In vitro* experiment demonstrated that *S. glabra* showed antibacterial effects through inhibiting the growth of *Streptococcus mutans* along with the activity of its glucosyltransferase (Huang and He, 2001). Besides, the aqueous extract of *S. glabra* could significantly promote the exosmosis of glucose and aspartate amino transferase in *Helicobacter pylori* and its drug-resistant bacteria at the concentration of 95 μg/ml, indicating that its antibacterial mechanism may be related to the damage of the outer membrane barrier (Guo, 2015). Some phenolic acids, coumarins and flavonoids isolated from the antibacterial fraction of *S. glabra* also showed good antibacterial activity (Wang and Ma, 1979b; Xu et al., 2008; Yuan et al., 2008). Fumaric acid and succinic acid had been proved to have excellent antibacterial effects on *S. aureus* and *P. aeruginosa* (Wang and Ma, 1979b). Isofraxidin (**187**) and 4,4′-bisofraxidin (**195**) showed good antibacterial effects on *Porphyromonas gingivalis* and *Streptococcus transglucosans* respectively, and their corresponding MIC values were 0.078 mg/ml and 0.125 mg/ml (Xu et al., 2008). Also, Kaempferol-3-O-β-D-glucuronide (**110**) exhibited a strong inhibitory effect on *S. aureus*, and its diameter of bacteriostasis circle was 14.67 ± 0.08 mm (Yuan et al., 2008). However, the current pharmacological studies mainly concentrate on *in vitro* models, and lack of discussion on the bioactive components and mechanism of antibacterial effect. Therefore, it is necessary to further evaluate the antibacterial effect and specific mechanism of *S. glabra* on *in vivo* models.

### Antiviral

*S. glabra* extract (250 mg/kg) could reduce the incidence rate and mortality of restraint stress mice caused by H_1_N_1_ influenza virus *via* reducing the pathological changes and the amount of virus in lung tissue, as well as regulating susceptibility genes and inhibiting the expression of pro-inflammatory factors (Cao et al., 2012). However, the dose used in this study was too high, and it could be considered to be reduced in future studies. What’s more, the ethanol extract of *S. glabra* could reduce pulmonary edema, inhibit viral replication in lung tissue and alleviate oxidative stress level in mice infected with H_1_N_1_ virus, and its mechanism may be related to activating nuclear factor-erythroid 2-related factor 2 (Nrf-2)/heme oxygenase-1 (HO-1) pathway to regulate superoxide dismutase (SOD), malondialdehyde (MDA) and NO to reduce oxidative stress injury (Huo et al., 2020). In recent years, it has been found that some components from *S. glabra* exhibit antiviral effects (Liu J.-x. et al., 2017; Wang et al., 2017). Rosmarinic acid-4-O-β-D-glucoside (**150**) could reduce the mortality of mice with pneumonia caused by A/FM/1/47 H_1_N_1_ virus at the concentration of 20 and 50 mg/kg (Liu J.-x. et al., 2017). Eleutheroside B1 (**193**) could inhibit the influenza virus ribonucleoprotein and the expression of RN mRNA (Wang et al., 2017). These results indicated that *S. glabra* has the potential to be developed as new drugs for the treatment of viral infectious diseases. Thus, in-depth research on active components and mechanism of antiviral activity should be taken into consideration.

### Anti-Inflammatory

*S. glabra* showed significant anti-inflammatory activity, which had a certain degree of inhibitory effect on various inflammation models. *In vitro*, Xie *et al.* confirmed that polysaccharide and ethyl acetate extracts from *S. glabra* could inhibit RAW264.7 cell proliferation and NO expression (Xie et al., 2010). Besides, studies have proved that sesquiterpenes, phenolic compounds and coumarins from *S. glabra* may be the bioactive components of its anti-inflammatory effect (Liu et al., 2015; Tsai et al., 2017; Wei et al., 2019). Wei *et al.* isolated ten sesquiterpenes from the anti-inflammatory fraction of *S. glabra* and found that all of them could inhibit NO production in RAW264.7 cells induced by LPS (Wei et al., 2019). Among them, shizukaol D (**67**: 5, 10, 15, and 20 μM) showed the most significant anti-inflammatory effect with IC_50_ values of 8.13 ± 0.37 μM, and its mechanism may be related to activating protein kinase B (AKT) to regulate Nrf2/HO-1 signaling pathway, thus down-regulating inducible nitric oxide synthase (iNOS) expression, inhibiting phosphorylated nuclear factor kappa B (NF-κB) expression along with nuclear translocation and regulating the activity of oxidation indexes (Wei et al., 2019). Furthermore, isofraxidin (**187**: 1, 5, and 15 mg/kg) had also been proven to improve the survival rate of mice induced by LPS *via* inhibiting the production of pro-inflammatory cytokines such as NF-κB, NO, interleukin-6 (IL-6) along with tumor necrosis factor alpha (TNF-α) and reducing the damage of inflammatory factors to organs. The mechanism may be related to the inhibition of TNF-α expression by regulating NF-κB signaling pathway (Liu et al., 2015). Therefore, *S. glabra* may play its anti-inflammatory effect mainly by regulating the expression of inflammatory factors such as NF-κB, NO, IL-6, TNF-α and the signal pathways related to inflammation, but how to regulate them is not completely clear and needs to be further explored.

### Anti-Tumor

*S. glabra* had been reported to inhibit the growth of gastric cancer, leukemia, liver cancer, lung cancer and other malignant tumors, which played an anti-tumor role by regulating cell cycle and inducing cell apoptosis. *Zhongjiefeng* injection, a Chinese patent medicine made from *S. glabra*, was reported to have a strong cytotoxicity on human lung cancer A-549, colon cancer HCT-29 and gastric cancer BGC-823, with IC_50_ values less than 50 μg/ml (Zhao et al., 2008). *Zhongjiefeng* tablets, made from *S. glabra*, could induce p21 expression by up-regulating TGF-β pathway, and arrested A549 and H1299 cells in G0/G1 phase, thus inducing cell apoptosis and inhibiting cell proliferation (Chen et al., 2018). The total flavonoids extract from *S. glabra* (25, 50 and 100 μg/ml) also showed significant inhibitory effect on leukemic K562 cells, which could promote cell apoptosis by decreasing the expression of Bcl-2 and caspase-3, and increasing expression of Cleaved caspase-3 (Sun et al., 2019). The polysaccharide from *S. glabra* (SGP-2) could inhibit human osteosarcoma cells U2OS proliferation and promote U2OS cells apoptosis at the concentration of 31.25, 62.5, and 125 nM, through down-regulating extracellular regulated protein kinases (ERK)/eIF4F/Bcl-XL signaling pathway to promote the release of cytochromes C and activate caspase protein (Zhang et al., 2014). Moreover, in S-180 cell-derived tumor mice model, it was further confirmed that SGP-2 (25, 50, 100 mg/kg) could inhibit the growth of transplanted tumor and activate endogenous apoptosis pathway through down regulating ERK-eIF4F pathway (Zhang et al., 2014).

### Immune Regulation

Jiang *et al.* reported that *S. glabra* could enhance the clearance index of macrophages in mice, but it had no obvious effect on specific humoral immunity, indicating that *S. glabra* mainly acted on the non-specific immunity of the body (Jiang et al., 2001). Meanwhile, *S. glabra* polysaccharide extract played an immune role through promoting the expression of membrane protein-related immune molecules and regulating the expression of pro-inflammatory and anti-inflammatory cytokines in RAW264.7 macrophages (Jiang et al., 2014). Furthermore, *S. glabra* also ameliorated immunodepression caused by stress. In restraint stress model mice, it was found that *S. glabra* extract (125 mg/kg) not only increased the number of lymphocytes, natural killer cells and natural killer T cells, normalized the ratio of T lymphocyte subsets, but also significantly reduced the lipid peroxidation level in spleen cells and increased the activity of oxygen free radicals, which partly through improving the ability of antioxidant to enhance immunity (He R. R. et al., 2009; He R. et al., 2009).

### Antioxidant

*S. glabra* extract exhibited strong free radical scavenging ability. Aqueous extract of *S. glabra* could scavenge hydroxy free radical in a concentration-dependent manner, at the concentration of 1.2 mg/ml, the scavenging rate on hydroxy free radical reached 89.89% (Qin et al., 2007). Aqueous extract of *S. glabra* also had a significant scavenging effect on DPPH radical, with half scavenging concentration of 13.49 mg/l (Li et al., 2009). *S. glabra* polysaccharide had obvious scavenging effect on hydroxy, superoxide anion, DPPH, and ABTS radicals (Jin et al., 2012). The active components of *S. glabra* also had the ability of scavenging free radicals. It was found that phenolic acids isolated from antioxidant active sites, such as rosmarinic acid (**149**), chlorogenic acid (**157**), and cryptochlorogenic acid (**159**), as well as flavonoids, such as quercetin-3-O-α-D-glucuronide (**114**) and neoastibin (**120**), showed antioxidant activity with strong ability of DPPH radical scavenging (Li et al., 2009, Li et al., 2010). In addition, ethanol extract, astilbin (**119**) and rosmarinic acid (**149**) from *S. glabra* had been reported to exhibit significant antioxidant activities, which could directly or indirectly scavenge reactive oxygen species (ROS) to protect mesenchymal stem cells from oxidative stress at the concentration of 10–100 μg/ml and hydroxy free radical mediated DNA damage at the concentration of 20–110 μg/ml. More importantly, the antioxidant capacity of ethanol extract from *S. glabra* may be related to the presence of total phenolics, especially astilbin and rosmarinic acid (Liu et al., 2016). These studies implied that *S. glabra* had the potential to treat a variety of diseases associated with oxidative stress. But, the current studies on antioxidant activity mainly focus on *in vitro* models, and a variety of *in vivo* models should be established to further evaluate its antioxidant activities, and to explore the relevant targets and pathways.

### Anti-Thrombocytopenic

Nowadays, *S. glabra* is commonly used to treat hemorrhagic diseases caused by thrombocytopenia, and its extract has been made into a Chinese patent medicine in China that are used to increase the platelets. Experimental studies had shown that *S. glabra* extract and its single drug preparation--*Xuekang* oral liquid could increase the number of peripheral blood platelets in mice with immune thrombocytopenic purpura, and the experimental results also showed that the total flavonoids from *S. glabra* (TFSG) was better than positive control (prednisone) in increasing the platelets (Xu et al., 2005). Besides, in bone marrow stromal cells-megakaryocyte co-culture system, TFSG (1.95, 3.9, and 7.8 μg/ml) promoted the differentiation and maturation of megakaryocytes in the co-culture system, which may be related to decreasing the rate of stromal cell apoptosis, regulating the content of cytokines that promote megakaryocyte differentiation including TPO, stromal cell derived factor-1 (SDF-1), TGF-β1, and vascular cell adhesion molecule-1 (VCAM-1), thereby affecting the state of stromal cells and secretion function. And the experimental results also suggested that this may be one of the mechanisms of *S. glabra* in the treatment of immune thrombocytopenia (Lu et al., 2019).

At present, most chemotherapeutic drugs can cause bone marrow suppression and lead to thrombocytopenia, *S. glabra* can significantly resist these side effects. Studies had shown that *S. glabra* significantly improved thrombocytopenia induced by 5-FU (Zhong et al., 2005). Based on this, Lu *et al.* established thrombocytopenia mice to explore the mechanism of TFSG on improving thrombocytopenia induced by chemotherapy (Lu et al., 2018a). The results demonstrated that TFSG (31.5, 63, and 94.5 mg/kg) could promote the secretion of TPO from stromal cells in the bone marrow microenvironment and the corresponding receptor C-mpl expression in megakaryocytes, then promote megakaryocyte to release mature platelets by regulating the TPO-C-mpl pathway. In addition, TFSG (31.5, 63, and 94.5 mg/kg) could also promote the proliferation, differentiation and maturation of megakaryocytes by promoting SDF-1 in bone marrow and the corresponding receptor CXCR-4 expression in megakaryocytes, thereby accelerating megakaryocyte to produce platelets (Lu et al., 2018b). These experimental results indicate that TFSG can promote megakaryocyte proliferation through multiple pathways and multiple targets, thereby increasing the number of platelets, but how does the TFSG promote the secretion of TPO or SDF-1 from stromal cells in the bone marrow microenvironment and regulate their corresponding receptors in megakaryocytes are still unclear, and further studies are needed to clarify.

### Hepatoprotective

*S. glabra* had significant protective effects on various liver injury models. In rat with liver injury induced by dimethylnitrosamine, *S. glabra* could significantly improve the pathological changes of liver tissue, and it not only normalized the serum protein index, but also enhanced the level of antioxidant index (Jin and Li, 1998). In mice with liver injury caused by P. acnes-LPS, the plasma alanine aminotransferase (ALT) activity increased, however, *S. glabra* extract could significantly reduce this trend, and the inhibition rate of high dose of the extract was up to 78.5% (Li et al., 2008). Meanwhile, 70% ethanol extract of *S. glabra* and seven sesquiterpenes from the extract showed significant hepatoprotective activity in hepatic epithelial stem cells from WB-F344 rats induced by D-galactosamine, among them, chloranoside A (**7**) and sarcaglaboside A-C (**25–27**) showed stronger liver protection activity than the positive drug dicyclool (Li et al., 2006a). Besides, *S. glabra* also had a good inhibitory effect on liver fibrosis. It was found that *S. glabra* extract reduced the serum liver function indexes (ALT and aspartate aminotransferase (AST)), liver fibrosis indexes (hyaluronic acid (HA), procollagen type III (PC-III), procollagen type IV (C-IV) and laminin (LN)) and tissue inhibitor of metalloproteinase-1 (TIMP-1) in rats with hepatic fibrosis induced by CCl_4_, as well as increasing the level of albumin (ALB). In particular, it could reduce the content of TIMP-1 to the normal level, and the related research indicated that the decrease of TIMP-1 expression contributed to the degradation of liver fibrosis, so its mechanism may be related to decreasing the expression of TIMP-1 (Xiong et al., 2015).

### Hypolipidemic and Hypoglycemic

*In vitro* and *in vivo* experiments, the polysaccharide from *S. glabra* showed excellent hypoglycemic effect. *In vitro*, the inhibitory effect of *S. glabra* polysaccharide (SEPR1) on α-glucosidase (IC_50_ = 49.01 μg/ml) was significantly stronger than that of acarbose (IC_50_ = 148.3 μg/ml). While in diabetic mice induced by HFD/STZ, SEPR1 (100 and 200 mg/kg) showed hypoglycemic effect by reducing fasting blood glucose levels and relieving the insulin resistance, which was better than that of positive control Acarbose (10 mg/kg) and Metformin (200 mg/kg). And the experimental results also indicated that SERP1 could increase the activity of antioxidant enzymes and decrease MDA level (Liu W. et al., 2017). In addition, total flavonoids from *S. glabra* reduced the levels of triglyceride (TG), total cholesterol (TC) and low-density lipoprotein (LDL-C) in serum of mice with hyperlipidemia, and the hypolipidemic effect of the high-dose total flavonoids was similar to that of positive control (Ji, 2012).

### Others

*S. glabra* also exhibited other pharmacological effects. Aqueous extract, ethanol extract, and essential oil from *S. glabra* could shorten the healing time of experimental fracture in rabbits, among which aqueous extract had the most significant effect in promoting fracture healing (Shi et al., 1980). This pharmacological study was consistent with the traditional use of *S. glabra* in the treatment of fractures, but the specific mechanism and effective components were still unclear. In addition, *S. glabra* had a protective effect on sport-injured skeletal muscle cells. In exercise-induced injury rats, it could be observed that the levels of SOD, catalase (CAT) and total antioxidant capacity (T-AOC) in the skeletal muscle and tissues of the rats decreased, the levels of MDA, creatine kinase (CK) and lactate dehydrogenase (LDH) increased, meanwhile, related inflammatory factors such as TNF-α, interleukin-18 (IL-18) and IL-1β levels increased. After the intervention of *S. glabra* polysaccharide, these indexes were significantly improved, suggesting that *S. glabra* polysaccharide could promote the repair and remodeling process of skeletal muscle structure after injury (Liu, 2015; Wang et al., 2020).

## Toxicity

From the long-term medicinal and edible history, it can be found that *S. glabra* is a kind of medicine food homology herb with good safety. Zhang *et al.* indicated that the maximum tolerance dose of aqueous extract of *S. glabra* in mice was more than 20 g kg^−1^·bw, without obvious genetic toxic effect, and there was no pathological damage in rats fed with the extract for 90 days at a dosage of 1.67, 3.33, and 5 g kg^−1^·bw (Zhang et al., 2016). These results were consistent with the findings of Xia *et al.* Xia et al. (1996) and Sun *et al.* Sun et al. (1998). In their studies, the results of the acute toxicity test, genetic toxicity test and teratogenicity test of aqueous extract of *S. glabra* were negative, suggesting that *S. glabra* had almost no obvious toxicity. However, these studies have only evaluated the toxicology of aqueous extract of *S. glabra*, and have not yet systematically evaluated the toxicology of its ethanol extract or other extracts. Therefore, future toxicological studies need more abundant experimental models, multiple types of *S. glabra* extracts or its active ingredients for further evaluation.

## Discussion and Prospect

As a traditional Chinese medicine, *S. glabra* has a long history of medicinal use and definite clinical curative effect. It is traditionally used to treat many diseases, including joint swelling and pain, sore throat, carbuncle, traumatic fracture, tumor, bleeding, *etc*. Because of its significant pharmacological effects, such as antibacterial, antiviral, anti-inflammatory, anti-tumor and anti-thrombocytopenic effects that are found in modern studies, *S. glabra* has attracted extensive attention. After decades of efforts by scholars, research on *S. glabra* has achieved certain results on chemical constituents and pharmacological effects. However, there is still a lot of work needs to be further explored. The future research of *S. glabra* can be considered from the following aspects:

Firstly, *S. glabra* has used as a folk medicine in China for more than 300 years, and a great quantity of folk empirical prescriptions with remarkable therapeutic effect also have appeared. Among them, the production technology of Miao nationality using *S. glabra* to treat traumatic fracture has been included in the list of National Intangible Cultural Heritage Protection. Nevertheless, research on the relationship between the traditional efficacy and its modern pharmacological activity has not yet been thoroughly investigated. Therefore, we should look for the potential pharmacological effects of *S. glabra* on the basis of its traditional application. For instance, “*Fen Lei Cao Yao Xing*”, an herbal medicine book written in the Qing Dynasty (AD 1906), recorded that *S. glabra* was used to treat rheumatic numbness, arthralgia and myalgia. Nevertheless, there is currently a lack of modern pharmacological studies of *S. glabra* on rheumatic arthritis. *S. glabra* has the effect of clearing heat and detoxification, which has a good reputation as “natural antibiotics” in folk, and is often used to treat infective inflammation caused by bacteria and virus in clinic, showing remarkable therapeutic effects. Modern pharmacological research has found that *S. glabra* possesses significant antibacterial, antiviral, and anti-inflammatory effects, which scientifically explains its heat-clearing and detoxifying effects. However, studies on antibacterial, antiviral, and anti-inflammatory effects of *S. glabra* are still in its infancy. Thus, more experiments are urgently needed to clarify its bioactive components and mechanism of action, in order to further establish the correlation between the traditional application and the modern pharmacological activity of *S. glabra*.

Secondly, more than 200 chemical constituents have been isolated from *S. glabra*, such as sesquiterpenes, flavonoids, phenolic acids, coumarins, lignans, anthraquinones, *etc.* However, related research on the pharmacological effects and targets of these components are still insufficient. There are relatively more studies on isofraxidin and rosmarinic acid, which have been used as markers to control the quality of *S. glabra*, but they are not only the characteristic chemical components in *S. glabra* (Alagawany et al., 2017; Majnooni et al., 2020), and whether they are the main active components of *S. glabra* remains to be confirmed. Therefore, the chemical constituents of *S. glabra* need to be further excavated in order to find more potentially active and specific compounds.

Thirdly, *S. glabra* has a good inhibitory effect on leukemia, gastric cancer, liver cancer and other malignant tumors. Ji *et al.* reviewed that *S. glabra* mainly played an anti-tumor role by inhibiting proliferation, inducing apoptosis, inhibiting telomerase activity and improving immune function (Ji et al., 2016). However, the active components, related targets and signaling pathways of its antitumor effects are still unclear. This suggests that the active components of antitumor effect may be polysaccharide, flavonoids, rosmarinic acid, isofraxidin, 3,3′-biisofraxidin, as well as atractylenolide Ⅲ, and the mechanism may be related to regulating ERK-eIF4F signaling pathway, along with apoptosis-related protein including Bcl-2, Bax and caspase-3. Nevertheless, the anti-tumor research on *S. glabra* is not comprehensive enough, its effective anti-tumor components and related mechanism still need to be further studied in the future.

Finally, *S. glabra* possesses an excellent anti-thrombocytopenic effect. In 2013, Dong *et al.* summarized the research advance of *S. glabra* on thrombocytopenia diseases, and found that the effective part of *S. glabra* against thrombocytopenia was total flavonoids, which could promote megakaryocytes proliferation to increase the platelets (Dong et al., 2013). However, how *S. glabra* regulated megakaryocytes proliferation was not discussed in their review. In this paper, we summarized the mechanism of *S. glabra* against thrombocytopenia, and found that total flavonoids of *S. glabra* could promote megakaryocytes proliferation through regulating the content of cytokines promoted megakaryocyte differentiation including TPO, SDF-1, TGF-β1 along with VCAM-1 and promoting the expression of SDF-1 and TPO in bone marrow microenvironment as well as their corresponding receptors CXCR-4 and C-mpl in megakaryocytes. Furthermore, *Xuekang* oral liquid, a single plant-based drug extracted from *S. glabra*, has a remarkable curative effect on primary and secondary thrombocytopenic purpura, thrombocytopenia caused by chemotherapy and radiotherapy, without side effects, which is a unique Chinese patent medicine for increasing the platelets in China (Xu et al., 1997; Shi, 2009). At present, research on *Xuekang* oral liquid mainly focuses on clinical trials, and there are few studies on its active components and mechanism. In addition, isofraxidin rather than flavonoids is stipulated as a marker by *Chinese Pharmacopoeia* to control the quality of *Xuekang* oral liquid, thus, the components of anti-thrombocytopenic effect still need to be further studied.

In summary, *S. glabra* with a long history and widely distributed resources, has been widely used for anti-bacterial, anti-viral, anti-inflammatory, anti-tumor, and anti-thrombocytopenia in clinic. *S. glabra* as an excellent traditional medicine for the sufficient experience in traditional medicine as well as remarkable curative effect, is also a kind of medicine and food homologous plant with great development potential, which is worthy of in-depth research and exploration in the field of medicine.

## Abbreviations

Akt, protein kinase B; ALB, albumin; ALT, Alanine aminotransferase; AST, Aspartate aminotransferase; C-IV, procollagen type IV; CAT, catalase; CK, creatine kinase; ERK, extracellular regulated protein kinases; HA, hyaluronia acid; HO-1, heme oxygenase one; IL-6, interleukin-6; IL-18, interleukin-18; iNOS, inducible nitric oxide synthase; MDA, malondialdehyde; NF-ҡB, nuclear factor kappa B; NO, nitric oxide; Nrf2, nuclear factor erythroid 2-related factor 2; PC-III, procollagen type III; ROS, reactive oxygen species; SOD, superoxide dismutase; SDF-1, stromal cell derived factor-1; TGF-β, transforming growth factor beta; TPO, thermoplastic polyolefin; TNF-α, Tumor necrosis factor alpha; VCAM-1, vascular cell adhesion molecule-1; LN, laminin; TIMP-1, tissue inhibitor of metalloproteinase-1; TG, triglyceride; TC, total cholesterol; LDL-C, low density lipoprotein; T-AOC, total antioxidant capacity; LDH, lactate dehydrogenase.
